# Degradation of p53 by Human *Alphapapillomavirus* E6 Proteins Shows a Stronger Correlation with Phylogeny than Oncogenicity

**DOI:** 10.1371/journal.pone.0012816

**Published:** 2010-09-17

**Authors:** Leiping Fu, Koenraad Van Doorslaer, Zigui Chen, Tutik Ristriani, Murielle Masson, Gilles Travé, Robert D. Burk

**Affiliations:** 1 Department of Microbiology & Immunology, Albert Einstein College of Medicine, Bronx, New York, United States of America; 2 Équipe Oncoprotéine, UMR CNRS 7100, École Supérieure de Biotechnologie de Strasbourg, Illkirch, France; 3 Departments of Pediatrics, Epidemiology & Population Health and Obstetrics, Gynecology & Women's Health, Albert Einstein College of Medicine, Bronx, New York, United States of America; McMaster University, Canada

## Abstract

**Background:**

Human Papillomavirus (HPV) E6 induced p53 degradation is thought to be an essential activity by which high-risk human *Alphapapillomaviruses* (alpha-HPVs) contribute to cervical cancer development. However, most of our understanding is derived from the comparison of HPV16 and HPV11. These two viruses are relatively distinct viruses, making the extrapolation of these results difficult. In the present study, we expand the tested strains (types) to include members of all known HPV species groups within the *Alphapapillomavirus* genus.

**Principal Findings:**

We report the biochemical activity of E6 proteins from 27 HPV types representing all alpha-HPV species groups to degrade p53 in human cells. Expression of E6 from all HPV types epidemiologically classified as group 1 carcinogens significantly reduced p53 levels. However, several types not associated with cancer (e.g., HPV53, HPV70 and HPV71) were equally active in degrading p53. HPV types within species groups alpha 5, 6, 7, 9 and 11 share a most recent common ancestor (MRCA) and all contain E6 ORFs that degrade p53. A unique exception, HPV71 E6 ORF that degraded p53 was outside this clade and is one of the most prevalent HPV types infecting the cervix in a population-based study of 10,000 women. Alignment of E6 ORFs identified an amino acid site that was highly correlated with the biochemical ability to degrade p53. Alteration of this amino acid in HPV71 E6 abrogated its ability to degrade p53, while alteration of this site in HPV71-related HPV90 and HPV106 E6s enhanced their capacity to degrade p53.

**Conclusions:**

These data suggest that the alpha-HPV E6 proteins' ability to degrade p53 is an evolved phenotype inherited from a most recent common ancestor of the high-risk species that does not always segregate with carcinogenicity. In addition, we identified an amino-acid residue strongly correlated with viral p53 degrading potential.

## Introduction

The *Papillomaviridae* have a circular genome of about 7900 bp. The genome typically contains seven or eight open reading frames (ORFs). The early genes E1 and E2 are involved in viral genome replication, while E6 and E7 have been shown to be oncogenes. The late genes L1 and L2 encode structural proteins that comprise the viral capsid. Nucleotide similarity across the L1 ORF has been used as a guide for viral classification [1], [2]. Members of the same genus, named according to the Greek alphabet, share more than 60% nucleotide sequence identity across the L1. Members of these genera are clustered into species comprised of distinct PV strains (commonly referred to as ‘types’) that are >10% different from any other previously defined type.

Compelling data implicate HPV as the long sought sexually transmitted agent of cervix cancer [3], the second most common cancer among women worldwide [4]. Indeed, alpha-HPVs have been detected in more than 90% of cervical cancer tissues [5]. The ability of high-risk alpha-HPV infections to progress to malignancy is due, in large part, to the expression of the E6 and E7 oncogenes (reviewed in [6], [7], [8]). The E6 proteins are about 150 amino acids long and contain 2 zinc finger domains. One of the first functions associating a mechanism of carcinogenicity of the HPV16 and HPV18 E6 proteins was their biochemical ability to degrade cellular p53 protein and inhibit p53 growth suppressor functions [9]. The p53 gene has pleiotropic activities and is inactivated in a majority of cancers (reviewed in [10], [11], [12]). Normally, p53 is activated in response to DNA-damaged stress signals and results in cell cycle arrest and/or apoptosis amongst other activities. Previous studies indicated that E6s from high-risk HPV types HPV16 and HPV18 significantly decreased the half-life of p53, while low-risk type HPV11 did not [13], [14]. Based on these observations, the ability to degrade p53 was assumed to be uniquely associated with, and a major cause of the oncogenicity by high-risk HPVs.

One important caveat in this generalization is the observation that HPV6 and HPV11 are proportionally more commonly found in condyloma accuminata and laryngeal papillomas. Whereas, HPV16 and HPV18 primarily infect the cervix, indicating a preferential tissue tropism difference between members of divergent alpha-HPV species groups [1], [2]. Given that differences in tissue tropism between the prototypical high- and low-risk types might account for the differences in E6 functionality based on ancient evolutionary niche adaptation and sequence divergence, we sought to determine the evolutionary origin of E6 mediated p53 degradation. We designed an *in vivo* screening assay to analyze representative HPV types from each of the 13 alpha-HPV species for p53 degradation. The data were then analyzed in the context of both the phylogenetic grouping and the oncogenic potential of the HPV types considered. Based on these data, we argue that the E6 ORF capability to degrade p53 was inherited from a most recent common ancestor (MRCA) of the alpha 5, 6, 7, 9 and 11 species groups. Nevertheless, this phenotype was subsequently acquired in a different lineage, not associated with cancer. This implies that p53 degradation by E6 is not sufficient to designate a virus as oncogenic. In addition, the evolved phenotype arising independently in two different lineages suggests that the p53 degradation phenotype might share amino-acid residues and/or domains not shared by the non-degraders. Through the use of sequence alignments and site-directed mutagenesis we identified a single amino acid position highly correlated with the potential to degrade p53. We show that context of changes in this amino acid is important in phenotype switching. These studies indicate that nonsynonymous changes in different lineages can have profound phenotypic affects.

## Materials and Methods

### Plasmid expression vectors

Templates for the E6 ORFs were either cloned from previously typed clinical specimens (HPV types 2, 10, 11, 16, 18, 30, 52, 53, 56, 62, 66, 70, 71, 73, 90 and 106) or obtained from cloned HPV genomes generously provided by E.-M. DeVilliers and H. zur Hausen (Deutsches Krebsforschungszentrum, Heidelberg, Germany; HPV types 6, 40); R. Ostrow (University of Minnesota, Minneapolis; HPV type 26); A. Lorincz (Digene Diagnostics, Silver Spring, MD; HPV types 31, 35); G. Orth (Institut Pasteur, Paris; HPV types 34, 39, 42, 54) and T. Matzukura (National Institute of Health, Tokyo; HPV types 58, 61). The E6 ORFs were amplified by PCR using a 5′ primer that included an HA-tag sequence in frame with the E6 ORF and engineered restriction enzyme digestion sites, as described below. HPV16 HA-E6 was cloned into the *Not*I-*Eco*RI sites, whereas all other HA-E6s were cloned into the *Age*I-*Eco*RI sites of the pQCXIN vector (BD Biosciences Clontech, San Jose, CA). The presence and orientation of the E6 ORFs were confirmed by sequencing. The reference variant of each type was chosen, except for HPV16 where an African-1 lineage E6 was cloned from a clinical sample (AF472508) [15]. Since we did not detect the HPV71 reference sequence in any of our molecular epidemiology studies, a clinical HPV71 isolate was used (AY330623). The p53 ORF was PCR amplified from a plasmid kindly provided by Dr. Liang Zhu (Albert Einstein College of Medicine) incorporating an HA-tag at the amino-terminus as described [16] and cloned into the *Hind*III-*Eco*RI sites of the pCR3 vector (Invitrogen Corporation, Carlsbad, CA).

### Cell lines and transfection

The 293T and C-33A cell lines were the generous gift of Dr. Liang Zhu. The C-33A cell line is an HPV negative cervical cancer cell line expressing a mutant p53 protein [17], [18] and 293T is a human embryonic kidney cell line with a high transfection efficiency that facilitates the study of ectopically expressed genes. The 293T cell line has high levels of endogenous p53 due to the expression of simian virus 40 (SV40) large T antigen, which stabilizes and inactivates the p53 protein. Both cell lines were maintained in Dulbecco's modified Eagle's medium (DMEM) with 10% fetal bovine serum (FBS). The 293T cell line was transfected using a calcium phosphate method [19]. The C-33A cell line was transfected using Lipofectamine Plus (Invitrogen Corporation, Carlsbad, CA) according to the manufacturer's recommendations. For the double immuno-fluorescence experiments, C-33A cells were transfected using the ExGen reagent (Eurogentec, Belgium).

### Western blot analysis

The proteasome inhibitor, MG132 (Calbiochem, San Diego, CA), was dissolved in DMSO (Sigma-Aldrich, Inc. St. Louis, MO) at a concentration of 10 mM. Twenty-four hrs after transfection, the cells were either treated with MG132 (10 uM final concentration) or DMSO for 16 hrs and the cells were then lysed in 300 ul RIPA buffer (Santa Cruz Biotechnology, Inc. Santa Cruz, CA) in a 60 mm plate. The protein concentration of cell lysates was measured using the Bio-Rad Protein Assay (Bio-Rad, Hercules, CA) according to the manufacturer's recommendations. Equal amounts of protein lysates for each cell line (40 ug for 293T and 20 ug for C-33A) were separated by SDS-PAGE and analyzed by Western blot as previously described [20]. The qualitative ability of each E6 to degrade p53 expression levels was scored by 2 investigators based on 3 independent experiments. The ability of the E6 protein from different types to degrade p53 was concordant in all experiments.

### Determination of p53 half-life co-transfected with HPV E6

293T cells were transfected with HA-p53 and E6 plasmids as described above. Cycloheximide (CHX, Sigma-Aldrich, Inc.), an inhibitor of protein synthesis in eukaryotic organisms, was added to the medium 36 hrs after transfection at 50 ug/ml final concentration. The cell lysates were collected at 0, 30, 60 and 120 mins after addition of CHX. Western blot analysis was performed as describe above. The Western blot results were scanned and the intensity of the HA-p53 and β-tubulin bands were quantified by ImageQuant v. 5.0. The half-life (t_1/2_) was extrapolated from the data corresponding to the time needed to decrease one unit of log_2_ (band intensity at time zero).

### Site-directed mutagenesis

Site-directed mutagenesis was performed using the GeneTailor Site-Directed Mutagenesis System (Invitrogen Corporation) according to the manufacturer's recommendations. All mutations were confirmed by sequencing the complete E6 ORF using flanking primers in the vector.

### Double immuno-fluorescence assay

The double immuno-fluorescence experiments were performed as described previously ([21]). Briefly, C-33A cells grown on coverslips were transfected with the indicated E6 plasmids. Twenty-four hrs later, cells were rinsed with PBS, fixed in 4% paraformaldehyde for 20 min at 20°C, and permeabilized for 5 min in 0.1% Triton X-100. Coverslips were incubated for 30 min in blocking buffer (DMEM, 10% FCS) and treated as described below. For simultaneous observation of the native p53 and HA-E6, cells were first incubated with polyclonal anti-p53 antibody (FL393, Santa Cruz Biotechnology) diluted 1/200 in blocking buffer, washed with PBS and then incubated with mouse monoclonal anti-HA-tag antibody diluted 1/3000; thereafter, cells were incubated with a goat anti-rabbit IgG antibody conjugated to Alexa Fluor 568 (Molecular Probes) diluted 1/2000, washed with PBS, incubated with goat anti-mouse IgG antibody conjugated to Alexa Fluor 488 (Molecular Probes) diluted 1/2000, washed with PBS, and stained for DNA with DAPI. Coverslips were mounted onto slides and visualized with a Zeiss fluorescence microscope.

## Results

### Establishment of an *in vivo* p53 degradation assay

**Figure 1:**
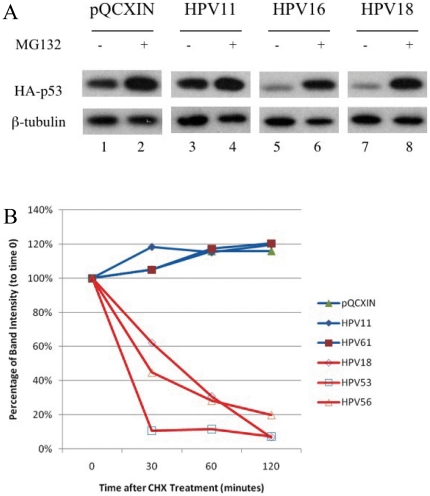
HPV E6-mediated p53 degradation. A) HA-tagged p53 levels were visualized by Western blot after co-transfection with HA-tagged HPV E6 from HPV11, HPV16 and HPV18 into C-33A cells. Lanes 1, 3, 5 and 7 show results from co-transfection of HA-p53 and vectors indicated at the top of the figure. Lanes 2, 4, 6 and 8 show the p53 levels after treatment with MG132, as indicated at the top of the figure. β-tubulin was visualized as a loading control. (B) Half life of HA-p53 in transfected 293T cells. 293T cells were transfected with HA-p53 and control (pQCXIN) or E6 ORFs (HPV11, HPV18, HPV53, HPV56 and HPV66). The band intensities were determined from the scanned Western blot using ImageQuant and the signals at time 0 were defined as 100%. The band intensities of the indicated time points were normalized to time 0.

p53 degradation is believed to play an important role in the oncogenic effects of E6 proteins from high-risk genital HPVs. In order to evaluate the E6 activity from a large number of HPV types, we designed a high-throughput *in vivo* p53 degradation assay. In this assay, the levels of ectopically expressed p53 in mammalian cells in the absence or presence of co-expressed E6 protein are evaluated by Western blotting. To validate the assay, we used three E6 proteins encoded by types with known activity for p53 degradation [9], [13] (Figure 1A). HPV11 E6 does not degrade p53 [13], whereas the E6 proteins from HPV16 and HPV18 have been shown to degrade p53 [22]. The three E6 ORFs were HA-tagged and co-expressed with HA-tagged p53 in C-33A cells. Compared to the empty vector control (pQCXIN), HPV11 E6 did not reduce the level of HA-p53, whereas HPV16 and HPV18 E6 reduced HA-p53 levels. Proteasomal inhibition with MG132 significantly increased the HA-p53 levels in cells expressing HPV16 and HPV18 E6, as compared to cells transfected with control vector or HPV11 E6 (figure 1A).

In order to show that the p53 degradation by HPV E6 in this assay was a post-translational event, we used the protein synthesis inhibitor cycloheximide (CHX) to estimate the half-life of p53 in the presence or absence of co-expressed E6. 293T cells were co-transfected with HA-p53 and E6 ORFs from HPV11, HPV18, HPV53, HPV56 or HPV61. The results showed that p53 half-life was decreased from greater than 2 hours (empty vector, HPV11, HPV61) to approximately 30 minutes (HPV18, HPV53, HPV56) (Figure 1B). Thus, E6 from HPV18, HPV53 and HPV56 decreased the levels of p53 via a post-translational effect, whereas E6 from HPV types 11 and 61 did not.

### E6-associated reduction in p53 levels is associated with phylogenetic grouping

**Figure 2:**
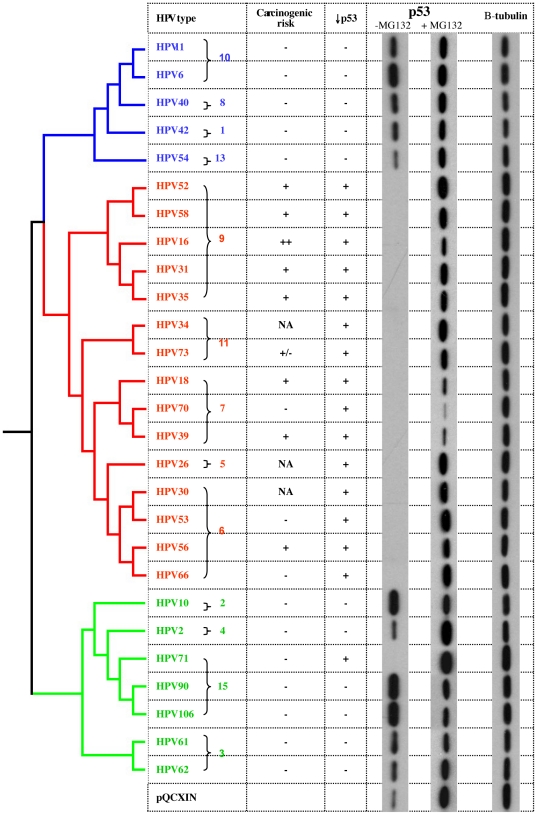
Phylogeny of alpha-HPVs and degradation of p53 by HPV E6 ORFs. HPV E6 activity on HA-p53 steady state levels were determined by Western blot using the assay shown in Figure 1A. The phylogenetic tree at the left was constructed using the combined early gene sequences as previously reported [35]. Epidemiological carcinogenicity was extrapolated from recent reviews [23], [24] and is indicated in the column labeled “carcinogenic risk”: ++, highly oncogenic; +, oncogenic; +/−, probably oncogenic; −, not significantly associated with cervix cancer; NA, insufficient data. The p53 levels after co-transfection with E6 from each type indicated on the left are shown in the far right column labeled, “p53” (with and without MG132) and the results are summarized in the column labeled“↓p53”. Endogenous β-tubulin (far right column) represents a loading control. The alpha-HPV species groups are indicated by brackets with a number to the right. The empty vector control, pQCXIN is shown at the bottom.

To evaluate the phylogenetic distribution of E6 activity to degrade p53, we tested E6 ORFs from representative types from each HPV alpha species group (Figure 2). Expression of the E6 protein from HPV types in species groups α9, α11, α7, α5 and α6 resulted in degradation of p53, whereas E6 from species α10, α8, α1, α13, α2, α4, α15 and α3 did not. An important exception was the observation that the E6 protein from HPV71 (α15) also reduced p53 levels.

These data demonstrated that the ability to decrease p53 levels was highly correlated with phylogenetic grouping and evolutionary descent. All the types that decreased p53 levels, except HPV71, share a most recent common ancestor (MRCA) (Figure 2). Nevertheless, it was noted that not all the E6 proteins that down-regulated p53 were from HPV types classified as group 1 carcinogens (i.e., sufficient evidence to be classified as carcinogenic to humans) by the Working Group of the WHO International Agency for Research on Cancer (IARC) [23], [24]. For example, HPV53 and HPV70 decreased p53 expression even though they are not epidemiologically associated with cervical cancer [5].

### Identification of a single amino acid associated with the E6's ability to degrade p53

**Figure 3:**
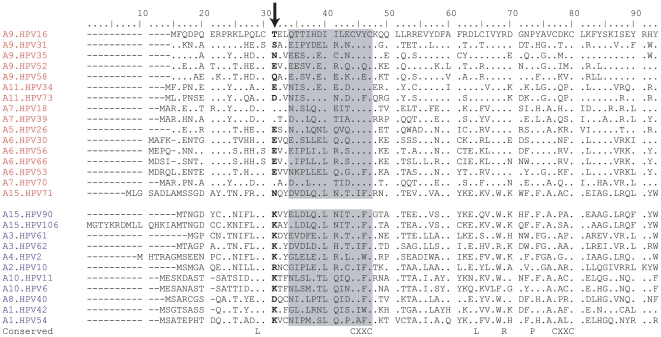
Alignment of HPV alpha E6 ORFs. The alignment of all 27 E6 ORFs tested in Figure 2 is shown. HPV types degrading p53 are shown at the top of the alignment and those not degrading p53 are shown at the bottom. The shaded region represents a proposed E6-AP binding domain [25], [26]. The amino acid sequences of the E6 ORFs were aligned using Clustal X (version 1.81) [36]. The amino acid at position 31 (arrow, **bold**) was associated with p53 degradation (p<0.01). “_” indicate gaps, whereas “.” indicates identical residues with the HPV16 E6 amino acid sequence shown in the top row.

Analyzing the p53 degradation potential in the context of phylogenetic relationships revealed that p53 degradation is highly correlated with the tree topology. This indicates an ancient lineage specific evolved trait in the MRCA of the alpha 5, 6, 7, 9 and 11 species groups and a more recent event in HPV71 (figure 2). Consistent with this notion, it should be possible to differentiate p53 degraders from non-degraders by the presence/absence of key amino acid residues and/or motifs. Based on an alignment of HPV types tested in this study (Figure 3), we identified a position (position 31, bold in the figure) at which the HPV types not able to decrease p53 (except HPV40) have a basic residue (lysine, K or arginine, R). In contrast, none of the E6 ORFs that degrade p53 have a basic amino acid at this site (p<0.01, χ^2^). This site is 2 amino acids upstream of a proposed E6-AP binding region (shaded in Figure 3) [25], [26]. HPV71, the only p53 degrader that did not cluster with the HR-HPVs, has an asparagine residue (N) at this site. Interestingly, HPV90 and HPV106, HPV71's closest relatives, have a ‘typical non-degrader’ lysine residue (K) at this site. This suggested a potential role for this amino acid in the degradation of p53 by E6.

The significance of this basic aa was investigated by identifying its structural location on the E6 protein. Although the complete structure of the E6 protein is currently unknown, the structure of the C-terminal domain of HPV16 E6 (E6C) has been determined [21]. Based on sequence similarities, it has been suggested that the fold of E6N domains should be comparable to that of E6C domains, and should be relatively conserved amongst different HPV types. Examination of the identified position (corresponding to HPV16 T17) in structure-based alignments of E6C and E6N domains [21] revealed that this residue aligns with a solvent-exposed position of the E6C domain situated at the N-terminus of the first helix of the domain [21]. Therefore, this residue may be involved in a protein-protein interaction interface in the E6/E6AP/p53 complex responsible for p53 degradation. Moreover, its exposed character makes it a suitable candidate for site-directed mutagenesis without expected changes to the structural integrity of the protein.

**Figure 4:**
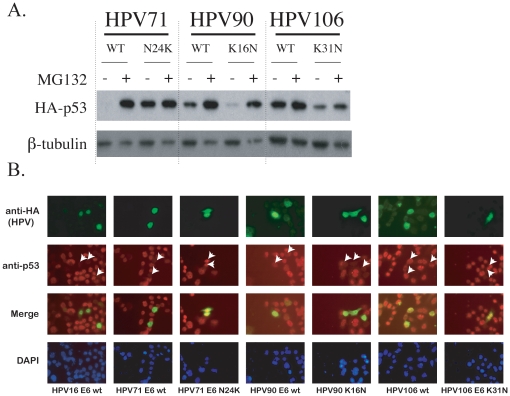
HPV E6 mutagenesis and degradation of p53. (A) The steady state levels of HA-p53 co-transfected with wild type or mutant HPV71, 90 and 106 HA-E6 in C-33A cells are shown in the row labeled HA-p53. The unmodified E6 ORFs are indicated with “WT” and the mutated E6 ORFs are indicated with the replacement amino acid at the top of the figure. β-tubulin is shown as a loading control in the bottom row. (B) Representative images of double immuno-fluorescence experiments. HA-tagged HPV E6 constructs are green, whereas the endogenous p53 is labeled red. In the p53 image, arrows indicate the location of the E6 expressing cells. Absence of p53 signal (red) is the result of degradation. In the merged images, yellow signifies a non-degrader, whereas green only (HPV E6) is indicative of a p53 degrader. Cell nuclei are detected with DAPI and are shown in the bottom row. Each HPV E6 construct is indicated at the bottom of each column.

To investigate the effects of “position 31”, we constructed single-point mutant E6 proteins: HPV71 E6 N24K, HPV90 E6 K16N and HPV106 E6 K31N. All E6 mutants were tested for their ability to degrade p53 using the Western blot assay. In parallel, the constructs were also tested for their ability to decrease p53 levels in a previously described single cell assay [21]. These mutations changed the activity of HPV71, HPV90 and HPV106 E6s (Figure 4A). The HPV71 N24K mutant completely abolished the p53 degradation ability of wt HPV71 E6. Whereas, wild type HPV90 E6 induced a slight MG132-sensitive decrease in p53 levels and a slight, but detectable decrease of p53 signal in single transfected cells (Figure 4B); the K16N mutation of HPV90 E6 enabled it to fully degrade p53 in Western blot-based assays (Figure 4A) and to turn off p53 signal in single transfected cell assays (Figure 4B) as efficiently as HPV16 E6. Wild type HPV106 did not decrease the p53 levels in either assay. The HPV106 E6 K-N mutant had weak activity for p53 degradation as assayed by immunoblot (Figure 4A), and did not induce p53 degradation in single transfected cells (figure 4B). Taken together, these data indicate a contribution of this position to p53 degradation.

## Discussion

Among the human *Alphapapillomaviruses*, a subset is considered oncogenic. The two major HR-types, HPV16 and HPV18 are responsible for about 70% of all cervical cancer cases [5], [27]. Recent work based on epidemiological and phylogenetic studies showed that all HR viruses cluster together in a HR-clade arguing that a common ancestor acquired an oncogenic potential (Fig 2) [28]. However, the most recent report by the working group of the IARC found that only a limited number of HPV types within this HR-clade had sufficient evidence to conclude that they were cervix cancer carcinogens [23], [24]. Nevertheless, there is compelling evidence supporting an evolutionary aspect to HPV induced carcinogenesis. In this study, we set out to investigate a well-characterized phenotype associated with the molecular basis of HPV oncogenicity from a phylogenetic perspective.

E6-mediated p53 degradation has been considered a hallmark function of oncogenic HPVs, although E6 is a highly multi-functional protein [7], [8]. The attribution of cancer risk being associated with proteasomal degradation of p53 has been mainly based on studies comparing low risk types HPV6/11 to high-risk types HPV16/18. These viruses, although all members of the *Alphapapillomavirus* genus have different tissue tropisms, cause different histological lesions (e.g., flat vs. verrucous) and are phylogenetically distant. These observations indicate that when extrapolating factors associated with cancer risk, evolution and biological niche adaptation (e.g., tissue tropism) need to be considered. In order to address the evolutionary importance of p53 degradation in the oncogenic process, we analyzed the ability of E6s from 27 HPV types (representing all alpha-HPV species) to degrade p53 *in vivo*. Our results demonstrate that although all high-risk types did in fact degrade p53, several low-risk types not epidemiologically associated with cervix cancer (e.g., HPV53, HPV70 and HPV71) also decreased p53 levels. A recent *in vitro* study [29] independently confirmed that E6 of HPV53, HPV56, HPV66 and HPV70 degraded p53 as efficiently as E6 of the highest-risk alpha-PV types. Interestingly, data from cutaneous HPV types (e.g., HPV5 and HPV8) associated with the development of non-melanoma skin cancer (NMSC) in the rare hereditary disease epidermodysplasia verruciformis (EV) [30], [31], [32] indicated that these viruses neither bind to, nor degrade p53. Therefore, the phylogenetic position of the virus is a more relevant criterion than its oncogenic risk for predicting ability to degrade p53. Based on these observations, we hypothesize that p53 degradation was adapted by the most recent common ancestor (MRCA) of the current HR-HPV clade in order to successfully adapt to a biological niche within the genital mucosa. All extant members of the HR-clade have inherited this p53 degradation phenotype irrespective of oncogenic potential. Embedded within this hypothesis is the notion that p53 degradation might be necessary, but is not sufficient for HPV induced oncogenesis. Thus, p53 degradation appears to be important for these viruses to complete their life cycle in this particular niche of the human body.

Furthermore, these results suggest that there are additional biochemical activities distinguishing closely related non-oncogenic (e.g., HPV53 and HPV70) from oncogenic members of the high-risk clade consistent with the plethora of activities associated with E6 [8]. However, since E6 and E7 expression is needed for successful transformation of primary keratinocytes [33], it is possible that the differentiating functionality lies within the E7 protein and/or an interaction of functions between the E6 and E7 proteins, introducing additional complexity into the biological system. Identifying specific changes associated with epidemiological cancer risk should lead to a better understanding of the oncogenic mechanisms utilized by high-risk HPVs that distinguish them from their phylogenetically closely related nearest neighbors.

Of note, HPV71 was one of the three most prevalent HPV types detected in a population based study in Costa Rica [34]. In addition, HPV71 is one of the types most likely to cause persistant cervicovaginal infection, but is not associated with neoplastic changes [28]. It is feasible that E6 activity to degrade p53 provides HPV71 an evolutionary advantage accounting for in part, its high population prevalence and persistence.

In an attempt to understand the evolutionary adaptations that occurred in the MRCA of the HR-clade, we identified a single amino acid site (“position 31”) correlated with the ability to degrade p53. This position, situated within the putative helix 1 of the E6N domain, is predicted to be fully exposed to solvent and therefore ideally positioned for participating in protein-protein interactions possibly important for p53 degradation. Both sequence analysis and mutagenesis data suggest that at this position a polar (e.g., T or N) or negatively charged residue supports p53 degradation activity, whereas a positively charged residue (R or K) is generally unfavorable to p53 degradation activity. This position may be part of an interacting surface domain important for the p53 degradation process. However, this interacting surface probably comprises several other amino-acid chains. Depending on the HPV type, the importance of position 31 may vary.

A structure based analysis of E6 in the light of the proposed evolution of the p53 degrading potential should allow researchers to further tease apart the evolutionary importance of position 31 for p53 degradation. Finally, whether E6 induced p53 degradation is a critical hallmark of HPV induced cancer or a manifestation of evolution remains to be confirmed. These data further support Theodosius Dobzhansky comment that “Nothing in biology makes sense except in the light of evolution.”
